# Integration Analysis of Transcriptome Sequencing and Whole-Genome Resequencing Reveal Wool Quality-Associated Key Genes in Zhexi Angora Rabbits

**DOI:** 10.3390/vetsci11120651

**Published:** 2024-12-13

**Authors:** Bohao Zhao, Yongqi Yu, Shaoning Sun, Jiawei Cai, Zhiyuan Bao, Yang Chen, Xinsheng Wu

**Affiliations:** 1 College of Animal Science and Technology, Yangzhou University, Yangzhou 225009, China; bhzhao@yzu.edu.cn (B.Z.); 13770249707@163.com (Y.Y.); 19952323987@163.com (S.S.); cjwcjw520800@163.com (J.C.); 18352764997@163.com (Z.B.); yangc@yzu.edu.cn (Y.C.); 2 Joint International Research Laboratory of Agriculture & Agri-Product Safety, Yangzhou University, Yangzhou 225009, China

**Keywords:** RNA sequencing, whole-genome resequencing, wool quality, Angora rabbit

## Abstract

Wool quality is a crucial economic trait in Angora rabbits, closely linked to hair follicle (HF) growth and development. Therefore, understanding the molecular mechanisms of key genes regulating HF growth and wool fiber formation is essential. In the study, fine- and coarse-wool groups were identified based on HF morphological characteristics of Zhexi Angora rabbits. According to the results, the diameters of fine and coarse fibers, and the percentage of coarse fibers, were significantly lower in the fine-wool group than in the coarse-wool group. Additionally, the HF density was higher in the fine-wool group than in the coarse-wool group, and the diameters of both primary hair follicles and second hair follicles were finer in this fine-wool group. Moreover, RNA sequencing (RNA-seq) and whole-genome resequencing (WGRS) were performed to identify key candidate genes and potential genetic variations between fine- and coarse-wool groups. RNA-seq analysis revealed 182 differentially expressed genes (DEGs), with 138 upregulated and 44 downregulated genes in the fine-wool group. The WGRS analysis identified numerous genetic variants including 15,705 InDels and 83,055 SNPs between the two groups. Additionally, the joint analysis of RNA-seq and WGRS showed enrichment of the Wnt, JAK-STAT, and TGF-β signaling pathways. The key overlapping candidate genes such as *DKK4*, *FRZB*, *CSNK1A1*, *TLR2*, *STAT4*, and *BMP6* were identified as potential crucial regulators of wool growth. In summary, this study provides valuable theoretical insights into wool quality and offers the potential for improving the molecular breeding of Angora rabbits.

## Simple Summary

Angora rabbits produce long, soft, and fluffy wool, which is a crucial economic trait for these animals. This study aimed to uncover the molecular mechanisms of key genes regulating HF growth and wool fiber formation in Zhexi Angora rabbits. The finding revealed that 182 genes in Angora rabbits, including 138 upregulated and 44 downregulated genes, were differentially expressed in the fine-wool group, as determined by RNA sequencing. Through whole-genome resequencing, numerous genetic variants were identified, including 15,705 InDels and 83,055 SNPs, distinguishing the fine-wool group from the coarse-wool group. Furthermore, a joint analysis of RNA-seq and WGRS highlighted candidate genes enriched in key signaling pathways, such as Wnt, TGF-β, PI3K-Akt, JAK-STAT, MAPK, and VEGF. Functional enrichment analysis identified key wool-growth-related genes, such as *DKK4*, *FRZB*, *TLR2*, *STAT4*, and *BMP6*. This study provides valuable insights and a reference for improving wool quality in Angora rabbits.

## 1. Introduction

Hair follicles (HFs) are unique, tiny organs in mammals that undergo periodic cycles, namely the anagen (growth phase), catagen (regression phase), and telogen (resting phase) [1]. Wool fibers originate from HFs embedded in the skin and are crucial commercial products for farm and wild animals, commonly harvested and used in the textile industry [2,3]. Unlike artificial fibers, wool fibers have diverse characteristics, such as heterogeneity and being pollution-free [4]. Angora rabbits, a special breed, produce long, soft, and fluffy wool. Their wool, known for excellent warmth retention, is often used in knitted goods and high-quality textiles. Angora wool ranks third among animal fiber products, after sheep wool and mohair, and is a significant raw material in the textile industry [5]. The future demands of the wool market require fine wool suitable for base-layer clothing, characterized by a fiber diameter of less than 18 μm, breathability, moisture-wicking properties, and odor resistance [6]. Furthermore, Angora rabbits produce medullated fibers with a fineness of 14–17 μm, which is finer than sheep wool [7]. This positions Angora wool as a valuable material for future consumer markets. German Angora rabbits are primarily raised for fine wool production, and a previous study showed that the average fiber diameter of German Angora rabbits in the northeast region of India was 19.9  ±  5.1 µm compared with those of the Chinese origin (11.8 µm), German origin (12.4 µm), and French origin (19.8 µm) [8]. With the increasing demand for fine wool from Angora rabbits, breeding for fine wool traits has become the dominating focus in Angora rabbit breeding programs. In the mid-1980s, German Angora rabbits were crossbred with the domestic Chinese Angora rabbits to develop a new variety in Zhejiang Province. This new breed, named Zhexi Angora rabbits, was officially recognized by the Chinese Domestic Animal Genetic Resources Committee. Zhexi Angora rabbits are now widely distributed across more than 20 provinces in China. However, few studies have been investigated the genetic improvement of Zhexi Angora rabbits [9].

Various factors, including breed, nutrition, climate, and management, affect rabbit wool quality. Studies have used low-coverage whole-genome sequencing to analyze the genetic architecture of wool traits and estimate quantitative trait loci (QTL) in Angora rabbits [10]. Using genome-wide markers, high-quality single-nucleotide polymorphisms (SNPs) were imputed in Angora rabbits to estimate the heritability of body weight and wool traits [11]. Whole-genome sequencing has also been used to uncover the genetic differences contributing to the long wool phenotype between Rex rabbits, New Zealand rabbits, and Angora rabbits, identifying 5.85 Mb regions with strong selective sweep signals in Angora rabbits [12]. Additionally, differentially expressed non-coding RNAs and mRNAs associated with various HF cycle stages were identified through RNA sequencing (RNA-seq) [13]. The RNA-seq analysis identified differentially expressed genes (DEGs) between the coarse- and fine-wool groups and revealed that extracellular matrix (ECM)-related genes, keratin (KRT) families, and keratin-associated proteins (KAPs) may regulate the fine fiber structure in Angora rabbits [14]. Further studies on RNA-seq between long-wool and short-wool rabbits have highlighted the importance of key signaling pathways, such as Hedgehog, Wnt, and TGF-β signaling pathways, in wool growth [15]. Wool fineness, a critical quality parameter, has been investigated in sheep breeds through whole-genome resequencing (WGRS) to reveal genes associated with wool fineness in fine or semi-fine wool and coarse-wool sheep breeds [16]. RNA-seq analyses comparing the skin transcriptome between the fine- and coarse-wool groups in sheep have identified DEGs that play key roles in HF metabolism and wool fiber diameter [17]. Non-targeted metabolomic and transcriptomic studies on Alpine Merino sheep have identified key molecules associated with keratin filament and skin development, further shedding light on wool fiber regulation [18]. In this study, HF-related parameters were compared between fine- and coarse-wool groups in Zhexi Angora rabbits. RNA-seq and WGRS were performed to identify candidate genes and key genetic variations influencing wool production. This study provides a valuable reference for molecular breeding aimed at improving wool quality in Angora rabbits.

## 2. Materials and Methods

### 2.1. Sample Collection

The ear, skin and wool samples were collected from Zhexi Angora rabbits sourced from Zhejiang Baizhongwang Angora Co., Ltd., Shaoxing, Zhejiang Province, China. The diameter of the coarse and fine fibers of the rabbits (*n* = 153) was manually measured using a projection microscope. The percentage of coarse fibers was manually estimated by calculating the ratio of the total weight of coarse and heterotypical fibers to the total weight of the 500 mg rabbit wool sample. Based on these measurements, six rabbits with the lowest fiber diameter and percentage of coarse fibers were selected for the fine-wool group, while six rabbits with the highest fiber diameter and percentage of coarse fibers were selected for the coarse-wool group. A 1 cm^2^ ear sample was collected from each rabbit for DNA extraction. Additionally, dorsal skin samples (1 cm^2^) were collected from the same position for histological analysis and RNA extraction. The skin samples were fixed in 4% paraformaldehyde for histological analysis, and paraffin sections were stained with hematoxylin–eosin (HE) for tissue observations. Total RNA was extracted from the skin samples by using the RNAsimple Total RNA Kit (Tiangen, Beijing, China). HF density (the number of HFs per mm^2^) was manually observed under an optical microscope, with six regions from each sample measured for both the coarse- and fine-wool groups. The diameters of primary hair follicles (PHFs) and secondary hair follicles (SHFs) were manually measured using the Mshot Image Analysis System V1.1.5, with six HFs from each sample analyzed. The experimental procedures for this study were approved by the Animal Care and Use Committee of Yangzhou University.

### 2.2. RNA Sequencing

For RNA-seq, dorsal skin samples were collected from the fine-wool group (n = 6) and coarse-wool group (n = 6, control group) of Zhexi Angora rabbits, The RNA’s purity and concentration were measured using a NanoDrop 2000 spectrophotometer (Thermo Scientific, Waltham, MA, USA), and the integrity of the total RNA was assessed using an Agilent 2100 bioanalyzer (Applied Biosystems, Carlsbad, CA, USA). RNA libraries were constructed, and 150-bp paired-end reads were generated using the Illumina NovaSeq platform. After evaluating the quality of the libraries, clean reads were mapped to the reference genome of *Oryctolagus cuniculus* (OryCun2.0) by using the HISAT2 tool [19]. Gene expression levels were quantified using fragments per kilobase of transcript per million (FPKM) mapped reads, and the differential expression analysis was performed using the DESeq2 package [20]. DEGs were identified with a fold change ≥ 1.5 and *p* < 0.05. All reads were deposited in the Short Read Archive of the National Centre for Biotechnology Information with the accession number PRJNA1155633.

### 2.3. GO Enrichment and KEGG Pathway Analyses

To elucidate the biological functions of DEGs, the Gene Ontology (GO) enrichment analysis was conducted using the clusterProfiler (3.8.1) R package. GO terms were categorized into biological processes (BPs), molecular functions (MFs), and cellular components (CCs), with gene length bias adjusted. KEGG pathway enrichment analysis was also performed using the clusterProfiler (3.8.1) R package to identify the enriched pathways of the DEGs.

### 2.4. Whole-Genome Resequencing

For the WGRS analysis, ear samples from both the fine-wool group (n = 6) and coarse-wool group (n = 6) were used to extract genomic DNA. These samples corresponded to the same individuals used in the RNA-seq analysis. DNA libraries for Illumina sequencing were constructed, and sequencing was performed on the Illumina HiSeq XTen platform. Raw reads in the fastq format were processed using fastp software for quality control. Subsequently, the clean reads were mapped to the rabbit reference genome by using BWA (Burrows‒Wheeler Aligner)-mem2 software [21]. The mapping results were sorted and converted to BAM files by using SAM tools software (v1.9) [22]. Following the mapping process, SNPs and InDels were identified using the HaplotypeCaller module in GATK (v3.8) [23] and filtered with the following criteria: QD < 2.0 || MQ < 40.0 || FS > 60.0 || QUAL < 30.0 || MQrankSum < −12.5 || ReadPosRankSum < −8.0, clusterSize 2, clusterWindowSize 5. On the basis of the reference genome, the identified SNPs were annotated using snpEff software [24]. Based on the mapping results, structural variation (SV) was detected using Manta software [25], and copy number variation was analyzed using FREEC software [26]. The WGRS data were deposited in SRA with the accession number PRJNA1156927.

### 2.5. qPCR with Reverse Transcription

The rabbit skin samples were collected, and total RNA was isolated from the samples by using the RNAsimple Total RNA Kit (Tiangen, Beijing, China). cDNA was synthesized using HiScript II Q Select RT SuperMix (Vazyme, Nanjing, China). The qPCR with reverse transcription (RT-qPCR) analysis was conducted using AceQ qPCR SYBR^®^ Green Master Mix (Vazyme, Nanjing, China) with a QuantStudio^®^ 5 instrument (Applied Biosystems, Waltham, MA, USA). The relative gene expression levels were analyzed using the 2^−ΔΔCt^ method [27], with glyceraldehyde 3-phosphate dehydrogenase (GAPDH) as the endogenous control. Primer sequences are listed in Table S1.

### 2.6. Joint Analysis of RNA-Seq and WGRS

Conjoint analysis was conducted by integrating the results of RNA-seq and WGRS. Overlapping genes between RNA-seq and WGRS analyses were identified by screening the common genes between the DEGs from RNA-seq and group-specific SNPs and InDels from WGRS. In addition, overlapping genes involved in HF growth and development-related pathways between RNA-seq and WGRS were identified based on KEGG pathway enrichment analyses of both WGRS and RNA-seq data.

### 2.7. Statistical Analysis

Statistical analyses were performed using SPSS 22.0 software (SPSS Inc., Chicago, IL, USA). GraphPad Prism 8 (GraphPad Software Inc., San Diego, CA, USA) software was used for generating graphical representations. All experiments included at least three biological replicates, and error bars in the results represent the mean ± SD. The threshold of *p* < 0.05 was considered statistically significant.

## 3. Results

### 3.1. Morphological Characteristics of HFs in Zhexi Angora Rabbits

**Figure 1:**
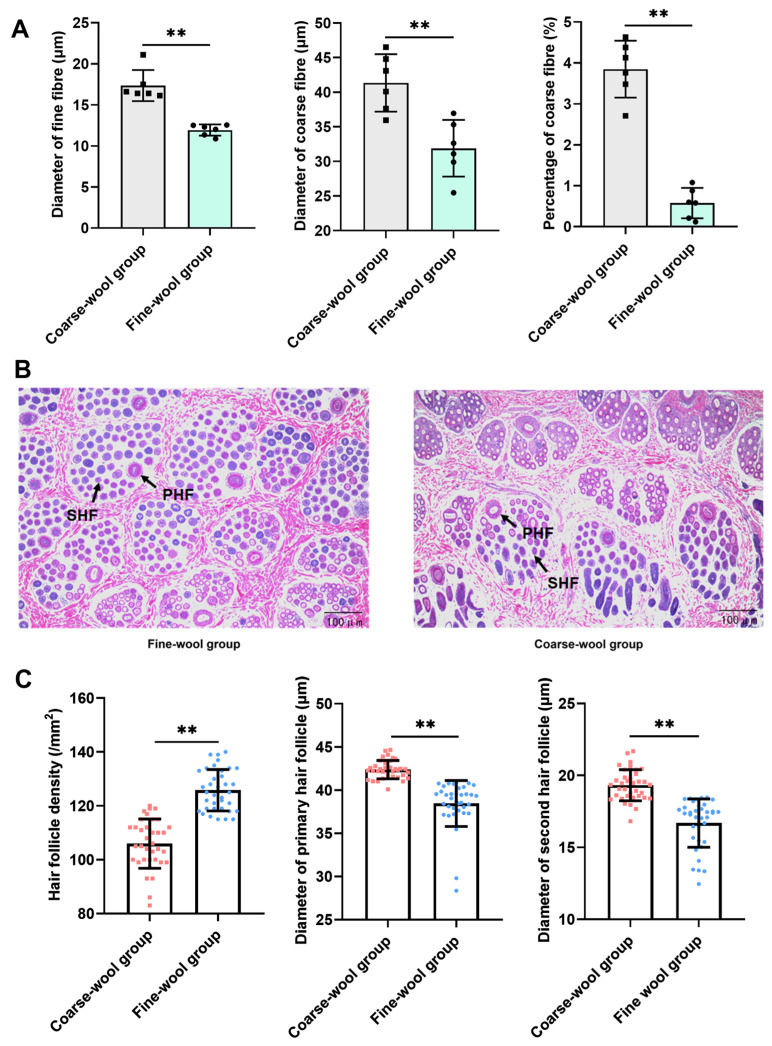
Morphological analysis between fine- and coarse-wool groups on the dorsal skin in Zhexi Angora rabbits. (**A**) The fine fiber diameter, coarse fiber diameter, and percentage of coarse fibers were measured between the fine- and coarse-wool groups in Zhexi Angora rabbits. (**B**) Histology of transverse sections of dorsal skin between the fine- and coarse-wool groups. (**C**) HF-related parameter analysis between the fine- and coarse-wool groups. PHF: primary hair follicle, SHF: second hair follicle. ** *p* < 0.01.

Wool samples were collected from Zhexi Angora rabbits, and the rabbits were divided into fine- and coarse-wool groups based on wool fiber traits. Phenotype measurements revealed that the diameter of fine fibers, diameter of coarse fibers, and percentage of coarse fibers were significantly lower in the fine-wool group than in the coarse-wool group (Figure 1A). Skin samples from the rabbits were collected from both groups, and the skin and HF morphology were observed through HE staining. The results demonstrated that the HF density was significantly higher in the fine-wool group than in the coarse-wool group (Figure 1B). Additionally, the diameters of the PHF and SHFs were significantly smaller in the fine-wool group than in the coarse-wool group (Figure 1C). These findings indicate that the fine-wool Angora rabbits had a higher HF density and produced finer wool fibers, suggesting the superior wool quality of these rabbits.

### 3.2. Screening of Fiber Formation-Associated DEGs Through RNA-Seq

**Figure 2:**
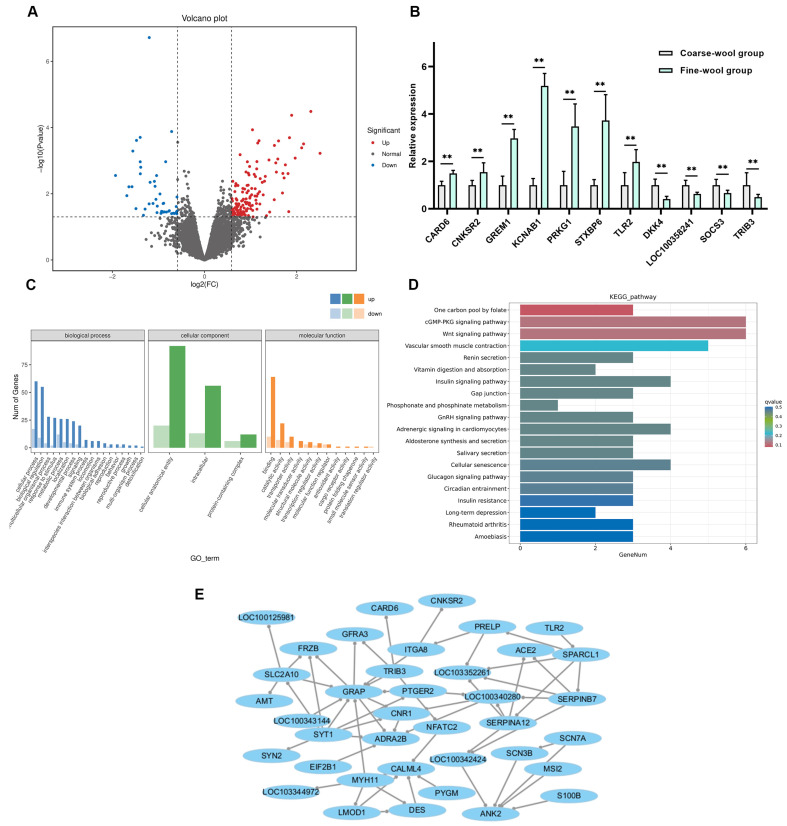
Screening of DEGs between fine- and coarse-wool groups in Zhexi Angora rabbits through RNA-seq. (**A**) Upregulated and downregulated mRNAs between the fine- and coarse-wool groups are shown in the volcano plot. (**B**) Validation of mRNA relative expression of DEGs between the fine- and coarse-wool groups through RT-qPCR. (**C**) GO enrichment analysis of DEGs between the fine- and coarse-wool groups. (**D**) KEGG enrichment analysis of DEGs between the fine- and coarse-wool groups. (**E**) The gene network of the DEGs was constructed using the STRING database. ** *p* < 0.01.

To identify key genes involved in wool fiber formation in Zhexi Angora rabbits, RNA-Seq analysis was conducted to detect DEGs between the fine- and coarse-wool groups. Table S2 presents the quality control and filtering information for raw data. A total of 182 DEGs were identified between the fine- and coarse-wool groups, with 138 upregulated and 44 downregulated genes (Figure 2A, Table S3). To verify the reliability of the RNA-seq results, seven upregulated DEGs (*CARD6*, *CNKSR2*, *GREM1*, *KCNAB1*, *PRKG1*, *STXBP6*, and *TLR2*) and four downregulated DEGs (*DKK4*, *LOC100358241*, *SOCS3*, and *TRIB3*) were randomly selected for validation through RT-qPCR. The results were consistent with those of the RNA-seq analysis (Figure 2B). Functional enrichment analysis of the DEGs was performed using GO and KEGG (Figure 2C,D). The DEGs were significantly enriched in GO terms related to processes such as cellular anatomical entity, cellular process, binding, biological regulation, and multicellular organismal process. Several GO terms directly associated with skin and HF development were enriched, such as regulation of keratinocyte proliferation, keratin filament, epidermal cell differentiation, and skin development. The KEGG signaling pathway analysis revealed that DEGs were involved in critical pathways associated with skin and HF development, including the Wnt signaling pathway, TGF-β signaling pathway, MAPK signaling pathway, PI3K-Akt signaling pathway, JAK-STAT signaling pathway, and VEGF signaling pathway. A gene interaction network was constructed using the STRING database, identifying 38 hub genes involved in functional associations between proteins (Figure 2E). Key DEGs, such as *FRZB*, *TRIB3*, *ACE2*, and *TLR2*, which are implicated in skin and HF growth and development, may play essential roles in wool fiber formation and growth.

### 3.3. Identification of Group-Specific Genetic Variation and Associated Genes Through WGRS

The WGRS of 12 samples from Zhexi Angora rabbits yielded a total of 1004.18 Gb of data. Quality assessment indicated a mean Q20 of 97.94%, mean Q30 of 94.54% (Table S4), and an average GC content of 42.46%. The average sequencing depth was 29× per individual, with an average genome coverage of 99.49%. The mapping rate of the samples aligned to the reference genome was 98.96%. Table S5 provides detailed information on genome coverage, sequencing depth, and other metrics for each sample. In total, 18,698,700 SNPs, 3,950,703 InDels, and 2,428,370 SVs were identified after mapping to the reference genome was completed using SAM tools. SNPs for the 12 samples included 4069 frameshift variants, 496 codon insertions, 398 codon deletions, and 64,200 synonymous and 35,330 nonsynonymous variants.

**Figure 3:**
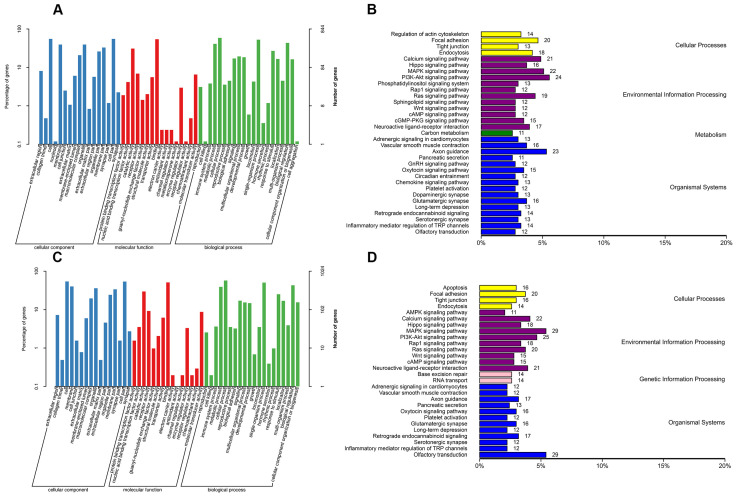
GO enrichment and KEGG pathway analyses of InDels and SNP sites located within genes. (**A**) GO enrichment analysis of InDel sites located within genes in CC, MF, and BP. (**B**) KEGG pathway enrichment analysis of InDel sites located within genes. (**C**) GO enrichment analysis of SNP sites located within genes in CC, MF, and BP. (**D**) KEGG pathway enrichment analysis of SNP sites located within genes.

Among the genetic variants between the fine- and coarse-wool groups, 15,705 InDels (Table S6) and 83,056 SNPs (Table S7) were identified. Notable InDels involved four codon changes and codon deletions in genes such as *ERGIC3*, *EVX2*, *SFRP5*, and *NACA*. Frameshift InDels were found in eight genes, namely *EPB41L1*, *MMP24*, *HM13*, *RPS23*, *LOC100354804*, *STARD9*, *ENSOCUG00000029040*, and *ENSOCUG00000031394*, and others. Codon-insertion InDels were detected in two genes, namely *NCOA6* and *ENSOCUG00000036372*. Among the SNPs, 326 synonymous and 171 nonsynonymous variants were identified in coding sequences between the fine- and coarse-wool groups, with 96 genes harboring nonsynonymous SNPs. GO enrichment and KEGG pathway analyses were used to explore the biological functions of group-specific InDels and SNPs (Figure 3A–D).

The GO terms significantly enriched were associated with HF growth and development, such as hair cell differentiation, skin development, negative regulation of the hair cycle, establishment of the skin barrier, and keratin filament formation. The KEGG pathway analysis highlighted candidate genes enriched in pathways critical to skin and HF development, including the Wnt signaling pathway, TGF-β signaling pathway, Hedgehog signaling pathway, Hippo signaling pathway, MAPK signaling pathway, and JAK-STAT signaling pathway. Key genes identified through functional enrichment, which may play essential roles in HF growth and wool quality traits include *SFRP2*, *SFRP5*, *CTNND2*, *TRPV3*, *CSNK1A1*, *SMAD7*, and *FERMT1*. These genes likely regulated wool fiber formation via molecular pathways and biological functions essential for HF development.

### 3.4. Joint Analysis of RNA-Seq and WGRS

To further elucidate the genetic basis of HF growth and wool fiber formation in Zhexi Angora rabbits, conjoint analysis was conducted by integrating the results of RNA-seq and WGRS. This analysis aimed to identify candidate genes that are crucial for regulating these traits. In total, 12 overlapping genes were identified between the DEGs from RNA-seq data and group-specific SNPs and InDels from WGRS (Table 1). Among these, the *KCNAB1* gene exhibited the most significant differences in DEGs, the *TLR2* gene had the largest fold change in expression between the fine- and coarse-wool groups, and the *PRKG1* gene had the largest number of SNPs and InDels in the overlapping gene group. Furthermore, the KEGG pathway enrichment analyses of both WGRS and RNA-seq data revealed common pathways involved in HF growth and development. The Wnt, TGF-β, PI3K-Akt, JAK-STAT, MAPK and VEGF signaling pathways were enriched in the RNA-seq and WGRS analyses. Several key genes enriched in these overlapping pathways were selected as candidate genes for HF growth and wool quality traits, such as *DKK4*, *FRZB*, *CSNK1A1*, *STAT4*, *GREM1*, *BMP6*, and *TLR2* (Table 2).

## 4. Discussion

Zhexi Angora rabbits, a breed resulting from crossbreeding local Chinese Angora rabbits with German Angora rabbits, are known for their giant body size, high wool yield, and superior wool quality. By 11 months of age, these rabbits can produce approximately 2 kg of wool after a 73-day growth period, with average wool lengths of 4.6 and 4.8 cm and wool diameters of 13.1 and 13.9 µm in males and females, respectively [9,12]. Studies have identified non-genetic factors affecting wool quantity and quality in Angora rabbits, including sex, birth conditions, harvesting season, and physiological state [7]. In this study, the Zhexi Angora rabbits were divided into fine- and coarse-wool groups, revealing significant differences in wool traits. The fine-wool group exhibited superior wool quality, characterized by finer wool fibers, a lower percentage of coarse fibers, and higher HF density.

The RNA-seq analysis was conducted between the fine- and coarse-wool groups to better understand the genetic background of wool growth. In a previous study, sheep with diverse fiber types exhibited differences in the PHF size and skin thickness between coarse- and fine-wool sheep. RNA-seq analyses identified 1994 DEGs associated with skin and HF morphology and development [28]. The transcriptome studies in Angora rabbits have identified DEGs involved in wool fiber diameter regulation, including transcription factors, KAP, and KRT families, and ECM-related genes [14]. The RNA-seq analysis of the DEGs between the short-hair and long-hair rabbits revealed that KRT families, ECM-related genes, collagen genes, and laminin genes affected rabbit wool growth [29]. During the HF cycle of Angora rabbits, DEGs during the anagen, catagen, and telogen were selected. Genes involved in the Wnt, MAPK, and TGF-β signaling pathways are also known to play crucial roles in skin development and the HF cycle [13]. The skin and HF growth and development-related DEGs between the fine- and coarse-wool groups were also identified. *DKK4* functions as a Wnt antagonist, influences Eda-independent development of secondary hair production, and plays a significant role in SHF differentiation [30]. As a secreted Wnt inhibitor, *FRZB* was expressed in DPC and impacted feather axis formation in chickens [31,32]. *STAT4* is the part of the JAK-STAT signaling pathway. *STAT4* gene expression was significantly correlated with alopecia areata [33]. Furthermore, gremlin 1 (*GREM1*) was expressed in the epithelial matrix and the dermal sheath cup during the anagen phase of HFs [34]. *GREM1* is suggested to be involved in hair follicle cycling. *BMP6* was differentially expressed during the HF cycle. BMP6 overexpression inhibited the telogen–anagen transition [35]. ACE2 was highly expressed in keratinocytes compared with fibroblasts and melanocytes in skin tissues. It was primarily expressed in differentiating keratinocytes and basal cells [36]. *TLR2* was positively expressed in the matrix and infundibulum of HFs. TLR2 was upregulated during the regenerative anagen phase, possibly mediating hair follicle stem cell activation through the regulation of BMP signaling [37,38].

Studies used low-coverage whole-genome sequencing to estimate the QTL and genetic factors associated with wool traits in Angora rabbits. The *FGF10* gene was identified as associated with fiber growth and diameter, providing a reference for Angora rabbit breeding [10]. The population structure, genetic diversity, genomic selection from different rabbit breeds, and wool-growth-related SNPs were analyzed through WGRS. This investigation revealed key genes associated with hair development [39]. In our study, the InDels and SNPs related to wool growth and HF development in Zhexi Angora rabbits were screened through WGRS. Several candidate genes with crucial roles in the Wnt signaling pathway and other regulatory mechanisms were identified. Among the candidate genes, the *SFRP2* gene plays a negative role in skin and HF development via the Wnt signaling pathway. This gene is highly expressed during the catagen phase and negatively regulates keratinocyte proliferation [40,41]. The *SFRP5* gene is associated with multicellular organism development and the Wnt signaling pathway [42]. *TRPV3* was enriched in the negative regulation of the hair cycle according to the GO analysis. *TRPV3* is also highly expressed in the hair shaft and inner root sheath and is associated with impaired formation of the hair shaft and hair canal [43]. *FERMT1* was involved in keratinocyte migration, and negative regulation of the timing of anagen and the canonical Wnt signaling pathway. In a previous study, homozygous sequence variants of FERMT1 were found to be correlated with skin disorders in various human families through exome sequencing [44]. *SMAD7* may regulate the TGF-β and BMP signaling pathways. In transgenic HFs, SMAD7 induction affected HF morphogenesis and differentiation. It also plays a role in the morphogenesis of sebaceous glands [45].

The conjoint analysis of RNA-seq and WGRS data was conducted to identify candidate genes related to skin development, HF growth, and wool fiber formation in Zhexi Angora rabbits. In total, 12 overlapping genes, including *TLR2*, *CARD6*, *CNKSR2*, *TRIB3*, and *PRELP* were identified. TLR2 is essential for hair cycle progression and hair follicle stem cell activation and is expressed in the early secondary hair germ lineage [38]. Upregulated TLR2 expression in the fine-wool group indicated that TLR2 can promote SHF differentiation. The NOD-like receptor (NLR) signaling-related gene *CARD6* was upregulated in the psoriatic epidermis and exhibited differential expression in keratinocytes and the epidermis [46]. In sheep, *CNKSR2* acted as the targeted gene of miR-21 and was expressed at different HF development stages, thereby regulating it [47,48]. In our study, *CARD6* and *CNKSR2* expression was upregulated in the fine-wool group, suggesting the role of *CARD6* and *CNKSR2* in wool fiber formation and HF development. TRIB3 overexpression induced TGF-β signaling, activated resting fibroblasts, and regulated dermal thickening and myofibroblast differentiation [49]. *TRIB3* was significantly upregulated in the coarse-wool group, which means that the *TRIB3* gene has the potential to lead to thicker and coarser hair. After PRELP was overexpressed in the skin, the thickness of the hypodermal fat layer decreased and interfered with dermal collagen fibrils [50]. Based on our study results, *PRELP* may hinder PHF morphogenesis and negatively regulate coarse fiber formation. Overlapping signaling pathways between RNA-seq and WGRS were identified. The candidate genes were enriched in several signaling pathways known to regulate HF morphogenesis, such as Wnt, MAPK, TGF-β, JAK-STAT, and PI3K-Akt signaling pathways [51,52,53,54,55]. Thus, candidate genes associated with skin development, HF growth, and wool fiber formation were identified through RNA-seq and WGRS. However, the molecular mechanism of candidate genes needs to be investigated in future, especially the group-specific SNPs and InDels, as they can be used for molecular marker-assisted selection for improving wool quality in Angora rabbit.

In summary, the study estimated the morphological characteristics of HFs between fine- and coarse-wool groups in Zhexi Angora rabbits. The RNA-seq analysis revealed specific mRNA expression profiles, and WGRS identified group-specific genetic variations and associated candidate genes. GO and KEGG analyses demonstrated that these candidate genes are significantly involved in the regulation of wool fiber formation and HF growth and development. The findings provide a deeper understanding of wool quality and wool production in Angora rabbits. Future research should focus on elucidating the molecular regulatory mechanisms of the identified candidate genes and their regulatory roles in wool fiber formation in Angora rabbits, which could lead to improved breeding strategies and wool production techniques.

## Figures and Tables

**Table 1:** Overlapping genes between RNA-seq and WGRS analyses.

Gene Name	Gene ID	*p*-Value	log_2_ Fold Change	Chromosome	SNP Number	Indel Number
*KCNAB1*	ENSOCUG00000007885	0.0001	1.0415	14		2
*TRIB3*	ENSOCUG00000007636	0.0002	−1.3960	4	7	1
*TLR2*	ENSOCUG00000003367	0.0010	1.3993	15	2	
*STXBP6*	ENSOCUG00000005144	0.0015	0.9784	17		1
*PRKG1*	ENSOCUG00000011473	0.0058	0.6725	18	1238	184
*ALDH1L2*	ENSOCUG00000006962	0.0060	−1.0734	4	1	
*CNKSR2*	ENSOCUG00000014660	0.0089	0.8258	X	1	
*PRELP*	ENSOCUG00000009707	0.0141	0.6867	16	2	
*CARD6*	ENSOCUG00000005374	0.0232	1.0014	11	1	2
*ANK2*	ENSOCUG00000010618	0.0265	0.6598	15	1	
*CDH19*	ENSOCUG00000017601	0.0461	1.3454	9		1
*CA10*	ENSOCUG00000002345	0.0495	0.6092	19	1	

**Table 2:** Genes in overlapping pathways between RNA-seq and WGRS.

Overlapping Signaling Pathways	RNA-Seq	WGRS
Wnt signaling pathway	*DKK4*, *FMNL1*, *FRZB*, *NFATC2*, *PLCB4*, *RSPO1*	*INVS*, *CSNK1A1*, *RSPO2*, *DAAM2*, *PRKCA*, *TCF7L2*
JAK-STAT signaling pathway	*FHL1*, *SOCS3*	*STAT4*, *BCL2L1*, *STAT5B*, *SOCS2*, *IL13RA1*, *SOCS6*, *PTPN11*, *SOCS7*, *AKT3*
MAPK signaling pathway	*GRAP*, *LOC100358241*	*PLA2G4E*, *ANGPT1*, *PDGFRL*, *PPM1B*, *PRKCA*, *WDR7*
TGF-β signaling pathway	*GREM1*	*TGIF2*, *BMP6*, *SMAD5*, *RBL1*, *LTBP1*
PI3K-Akt signaling pathway	*ITGA8*, *LOC100353654*, *TLR2*	*PPP2R3A*, *COL6A6*, *BCL2L1*, *CREB5*, *COL4A3*, *TLR2*, *ANGPT1*, *PRKAA2*
VEGF signaling pathway	*NFATC2*	*PLA2G4E*, *PRKCA*, *PLA2G4F*, *PPP3CA*

## Data Availability

The datasets generated and analyzed during the current study are available in the NCBI repository, https://www.ncbi.nlm.nih.gov/bioproject/PRJNA1155633 (accessed on 10 December 2024) and https://www.ncbi.nlm.nih.gov/bioproject/PRJNA1156927 (accessed on 10 December 2024).

## Supplementary Materials

The following supporting information can be downloaded at https://www.mdpi.com/article/10.3390/vetsci11120651/s1, Table S1. Primers used for RT-qPCR; Table S2. The information of quality evaluation and filtration of raw data through RNA-seq; Table S3. The information of DEGs between the fine- and coarse-wool groups in Zhexi Angora rabbits; Table S4. The quality control of WGRS; Table S5. The mapping information of WGRS; Table S6. The information of InDels between the fine- and coarse-wool groups in Zhexi Angora rabbits; Table S7. The information of SNPs between the fine- and coarse-wool groups in Zhexi Angora rabbits.
